# Man vs Machine: Predicting First‐Pass Recanalization After Endovascular Thrombectomy

**DOI:** 10.1161/SVIN.123.000836

**Published:** 2023-05-02

**Authors:** Aayushi Garg, Edgar A. Samaniego

**Affiliations:** ^1^ Department of Neurology University of Pittsburgh Medical Center Pittsburgh PA; ^2^ Departments of Neurology Neurosurgery and Radiology University of Iowa Carver College of Medicine Iowa City IA

**Keywords:** Editorials, artificial intelligence, clot, large vessel occlusion, non‐contrast head computed tomography, radiomics, thrombi

The book edited by Martin H. Greenberg and John Helfers titled *Man vs Machine* is a collection of 15 stories that envision a world threatened by sophisticated technology, cornering humankind to a more limited role.^1^ As our lives become more and more entangled with technology, human performance faces competition from machine learning (ML), even in the practice of stroke care. Take, for example, the ability to predict clinical outcome after endovascular thrombectomy (EVT). No current algorithm or imaging biomarker can accurately predict the efficacy of EVT in leading to a good clinical outcome.^2^ Bala et al report in this issue of S:VIN, an interesting comparison of the human prediction of clinical outcome with the prediction derived from ML. The authors performed a post‐hoc analysis of the randomized controlled trial ESCAPE‐NA1 (Efficacy and Safety of Nerinetide for the Treatment of Acute Ischemic Stroke).^3^ The first pass effect (FPE) in patients undergoing anterior circulation EVT was analyzed. Radiomic features (RFs) were extracted from thrombus characteristics derived from baseline noncontrast computed tomography (NCCT) and CT angiography and used to build an ML model to predict FPE. This ML prediction model was compared with the FPE prediction of stroke experts.

Radiomics is a rapidly growing field that focuses on extracting quantitative data from medical images. RFs are mathematical descriptors of the texture, shape, and intensity of the pixels within an image, which can be used to identify patterns and predict outcomes. By applying ML algorithms to these RFs, researchers and clinicians can identify subtle changes that may not be visible to the naked eye. Radiomics has the potential to revolutionize the field of personalized medicine, as it can predict treatment response and improve patient outcomes. Bala and other authors have explored the use of radiomics in the analysis of thrombus in stroke patients who undergo NCCT and CT angiography as part of their stroke care.^4^, ^5^, ^6^, ^7^

The FPE is defined as complete recanalization of the large vessel occlusion and the downstream vasculature achieved in a single thrombectomy device pass.^8^ Among cases where complete recanalization is achieved, FPE is associated with improved clinical outcomes, independent of the time to recanalization.^9^ Multiple passes may be associated with higher risk of vessel injury, distal microembolization, and longer ischemia times, hence affecting clinical outcomes.^9^ Several patient‐ and procedure‐related factors can influence FPE. These include patient demographics, extracranial and intracranial vascular anatomy, thrombus characteristics and location, device type and procedural techniques, among others.^10^ The detailed characterization of thrombus perviousness, composition, and dimensions could guide treatment and device selections and may be a step toward improving FPE. Radiomics‐based image analysis may predict FPE more reliably because it can process multiple imaging characteristics that are not detected in conventional imaging.^11^ Bala et al showed that a radiomics‐based ML model was superior to traditional imaging characteristics in the prediction of FPE, even when compared with the assessments of stroke experts who analyzed all the clinical and imaging information. This study is based on a large clinical trial data set with uniform clinical and imaging information. However, as the authors acknowledge in the limitations section, the study did not account for key factors that may influence FPE, such as the experience level of the neurointerventionalist performing EVT.

Radiomics is far from being fully automated. To conduct their analysis, it was necessary for Bala et al to manually segment each thrombus. This process is time consuming and subjective, as thrombus boundaries must be determined manually, a process that can be hindered by the image resolution on NCCT and CT angiography. Moreover, it is often challenging to determine the presence of thrombus with NCCTs and to accurately establish the region of interest on a voxel‐by‐voxel analysis. Indeed, 4 experienced readers were trained to perform manual segmentation for 15 patient cases, followed by a feedback session to discuss the segmentation strategy. Similar manual segmentation of the thrombus has been described by others in performing RF analysis.^5^, ^6^ Therefore, although radiomics provided multiple metrics, the main step of the entire process – selecting the region of interest – must be performed manually, which can introduce bias.^12^ Despite the required manual processing, it is exciting that ML models based on RFs predicted more accurately clinical outcomes than stroke experts who used traditional imaging characteristics and clinical information. Besides predicting FPE, radiomics models have also been recently evaluated for the analysis of thrombus characteristics, such as thrombus composition. A recent study from Santo et al, compared the histology of 10 clots extracted through EVT and the radiological findings on pre‐EVT NCCT and CT angiography, complemented with a micro‐CT analysis that achieved a voxel size resolution of 4.9 μm.^7^ The global texture of the thrombus correlated with radiomic thrombus signatures, that could potentially be used to predict FPE. The detailed perviousness characterization of thrombus could lead to the prediction of response to tPA, or choice of device for hard thrombus. This preprocedural information could also help the prediction of possible thrombus fragmentation in case of red blood cell‐rich thrombus, and the presence of underlying atherosclerotic plaques in patients with intracranial atherosclerosis. The goal of this approach is a personalized assessment of thrombus composition to achieve the best possible outcome.

Even though the current radiomics‐based model showed better accuracy in predicting FPE than the conventional imaging features or the assessment by stroke experts, the area under the curve was 0.74, with a sensitivity of 79% and specificity of 60%. As the authors discuss, the performance of this model was slightly inferior to that reported in prior similar studies.^5^, ^6^ A more stringent reperfusion metric (TICI 2c–3) was used in this study, which may explain the lower accuracy compared with previous studies. The RFs derived from this ML model need to be validated in an external data set to test the robustness and stability of the model. External validation can help verify that model performance is not impacted by unexpected factors. This is of utmost importance for imaging‐based models, as interscanner and intervendor variability of features is important in radiomics.^13^ In cases in which radiomic studies rely on data from multiple scanners, neglecting this variability can jeopardize the replicability and reproducibility of the RFs. ESCAPE‐NA1 is a multicenter study that included 48 acute care hospitals in 8 countries. This is one of the strengths of the study by Bala et al, as imaging data were acquired in multiple scanners. However, the RF analysis and ML‐derived model should be validated in other cohorts. If the proposed radiomic‐based prediction model performs adequately on external data sets is yet to be determined.^12^

Future studies should focus on deriving and validating a prediction model for FPE that combines radiomics with other clinical and procedure‐related factors. A recent meta‐analysis showed that conventional ML models did not exhibit significant superiority when compared with prognostic scores for large vessel occlusion outcome predictions.^14^ In addition, more work is needed to automate the process of segmenting thrombus. Despite its promise, radiomics is still in its early stages, and further research is needed to validate its clinical utility. Nonetheless, the potential for radiomics to improve patient care and inform treatment decisions is significant, and it is an area of active research and development within the medical community.

*Man vs Machine* explores the potential benefits and drawbacks of ML and artificial intelligence. While these technologies offer significant potential for improving various aspects of our lives, such as healthcare, education, and transportation, they also raise concerns about privacy, bias, and accountability. For the time being, humans have not been replaced in predicting FPE outcomes and have a potential strong ally in radiomics in the quest for personalized medicine.

## Sources of Funding

None.

## Disclosures

None.
